# Role of the Carbon Nanotube Junction in the Mechanical Performance of Carbon Nanotube/Polyethylene Nanocomposites: A Molecular Dynamics Study

**DOI:** 10.3390/nano14060520

**Published:** 2024-03-14

**Authors:** Xian Shi, Xiaoqiao He, Xuefeng Liu

**Affiliations:** 1 School of Civil Engineering, Suzhou University of Science and Technology, Suzhou 215009, China; 2 Department of Architecture and Civil Engineering, City University of Hong Kong, Tat Chee Avenue, Hong Kong; 3 Center for Advanced Structural Materials, City University of Hong Kong Shenzhen Research Institute, Shenzhen 518057, China; 4 College of Hydraulic and Environmental Engineering, China Three Gorges University, Yichang 443002, China; 5 Department of Mechanics and Aerospace Engineering, Southern University of Science and Technology, Shenzhen 518055, China

**Keywords:** CNT, polymer, nanocomposite, molecular dynamics, mechanical performance

## Abstract

Carbon nanotube (CNT)-based networks are promising reinforcements for polymer nanocomposites without the issue of CNT agglomeration. In this study, the CNT junction, a vital and representative structure of CNT-based networks, was applied as the reinforcement of the polyethylene (PE) matrix. The tensile properties of the CNT-junction/PE nanocomposite were investigated via molecular dynamics (MD) simulations and compared with those of pure PE matrix and conventional CNT/PE nanocomposites. The CNT junction was found to significantly increase the mechanical properties of the PE matrix. The Young’s modulus, yield strength, and toughness rose by 500%, 100%, and 200%, respectively. This mechanism is related to the enhanced interfacial energy, which makes the polymer matrix denser and stimulates the bond and angle deformations of the polymer chains. Furthermore, the CNT junction demonstrated a more profitable reinforcement efficiency compared to conventional straight CNTs in the PE matrix. Compared to the ordinary CNT/PE model, the improvements in the Young’s modulus and toughness induced by the CNT junction were up to 60% and 25%. This is attributed to the reduced mobility induced by the geometry of the CNT junction and stronger interfacial interactions provided by the Stone–Wales defects of the CNT junction, slowing down the void propagation of the nanocomposite. With the understanding of the beneficial reinforcing effect of the CNT junction, this study provides valuable insights for the design and application of CNT-based networks in polymer nanocomposites.

## 1. Introduction

In recent decades, there has been a popular trend towards the development of lightweight and intelligent nanocomposite materials, for which a polymer-based nanocomposite material is an important aspect. A carbon nanotube (CNT) is considered to be an ideal filler material for polymer nanocomposites [1] due to its excellent mechanical and electrical properties [2,3]. To date, CNT-based polymer nanocomposites have received considerable attention in many fields, including flexible electronic devices [4], biomedical manufacturing [5], and electromagnetic interference shielding materials [6].

To uncover the underlying reinforcing mechanism of the CNT and achieve an effective design of the CNT/polymer nanocomposite, a series of theoretical investigations have been conducted. Various methods ranging from the macroscale to the nanoscale have been applied. Alian et al. [7] applied different multiscale modeling schemes to investigate the interfacial and mechanical properties of epoxy nanocomposites. Liu et al. [8] conducted coarse-grained molecular dynamics (MD) simulations for the CNT-fiber/polymer nanocomposite and explored its self-densified microstructure and enhanced properties. Kumar [9] developed a multi-scale finite element method to simulate the effect of CNT agglomeration in CNT/polymer nanocomposites.

Among these theoretical approaches, MD simulation is an effective method that can provide nanoscale insights for understanding the performance of polymer nanocomposite. There have been extensive studies applying the MD method to evaluate various performances of the resulting composite materials [10,11,12]. With MD simulations, a variety of impact factors have been effectively discussed in previous studies, such as the temperature [13], the loading rate [14,15], the geometry parameters, and the functional group of CNTs [16,17]. According to the pull-out simulations via the MD method, the reinforcing mechanism of CNTs has been uncovered as improved interfacial characteristics between the CNTs and the polymers [18,19]. Moreover, the MD simulations make it clear in the nanoscale that the key to effective reinforcement is to avoid an agglomeration of CNTs in the polymer matrix [20,21].

Compared to being individually distributed in the polymer matrix, CNT or graphene would have much higher reinforcing efficiency if they were connected as ordered networks. In the experimental studies, many researchers successfully composited polymers with different CNT or graphene networks. In the study of [22], the obtained graphene foam/conductive polymer composites were ultralight and able to achieve exceptional electromagnetic interference shielding. Ma and coworkers [23] prepared the polymer nanocomposites reinforced by CNT foam and concluded that CNT foam exhibited excellent adsorption performance for liquid polymer. Zhao et al. [24] produced a novel high-energy-capacity and multi-responsive phase-change fibers polymer via in situ polymer composition with expanded CNT networks.

However, there are only very limited theoretical studies focusing on the polymer nanocomposite reinforced by CNT-based networks. These studies are mainly about the polyethylene (PE) nanocomposite reinforced by pillared graphene networks, which is a novel CNT/graphene hybrid network. One study uncovered that the connection between the CNT and graphene leads to more mechanical interlocks between polymer chains, improving the interfacial performance and the synergistic effect of the nanocomposite [25]. Another study explored the effect of the structure, arrangement, and dispersion of pillared graphene on the tensile mechanical properties of PE nanocomposites [26]. Apart from that, in the study of [27], an analytical model was proposed to forecast the temperature-dependent tensile strength of CNT/polymer nanocomposites, and the effects of the CNT network were considered. It was concluded that forming the network structure is beneficial to improving the tensile strength of CNT/polymer nanocomposites.

For CNT-based networks, the most typical and common element must be the CNT junction, which is a branched structure produced by the connection of several CNTs. An experimental investigation concluded that branched CNTs employed as nanofillers in the polymer matrix are desirable in terms of both the elastic and dissipative properties [28]. Nevertheless, related theoretical investigations about CNT junctions are limited. There is a study that discussed the geometrical effect of nanoparticles on the structure, dynamics, and anisotropic viscosity of PE nanocomposites [29]. The X-shaped and Y-shaped CNT junctions were proven to have a relatively powerful nucleating ability, effectively increasing the entanglement densities of the PE matrix. In another study, the CNT junction was concluded to result in a more ordered structure and a larger spatial extension of the PE matrix [30].

Compared with the considerable experimental outcomes of the polymer nanocomposite reinforced by CNT-based networks, the above theoretical studies are far from enough. Further theoretical studies are needed to systematically clarify the reinforcing efficiency of the CNT junction and the corresponding mechanism. In this study, we investigated the role of the CNT junction on the mechanical properties of the polymer nanocomposite. A Y-shaped CNT junction was added to the PE matrix, and MD simulations were conducted. By comparing the pure PE matrix and the PE nanocomposite reinforced by ordinary CNTs, the reinforcing efficiency of the CNT junction was identified, and the underlying mechanisms are discussed.

## 2. Materials and Methods

**Figure 1:**
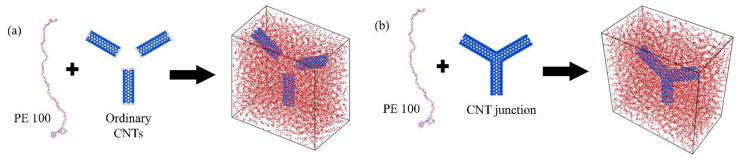
Two CNT/PE models with different reinforcements. (**a**) Ordinary disconnected CNTs; (**b**) CNT junction.

In this study, three full-atom models were established for MD simulations, including a pure PE model and two CNT/PE nanocomposite models. For the two CNT/PE nanocomposite models, the CNT reinforcements were different, which are presented in Figure 1. It can be seen that one model includes three ordinary CNTs and the other one is a Y-shaped CNT junction that is connected by three ordinary CNTs. Building the ordinary CNT/PE nanocomposite model in this specific way, instead of using just one straight CNT, was aimed at having a better comparison with the CNT-junction model.

The single-walled CNT, with a chirality of (6,6) and an aspect ratio of about 3.0, was used to form two different CNT reinforcements. The CNT Y-junction was fabricated by connecting three same single-walled (6,6) CNTs. According to previous studies on the CNT connection [31,32], six heptagonal atomic carbon rings were introduced in the connection area. All CNTs in the two models had hydrogenation at the end of the tube to avoid the influence of unsaturated bonds. PE chains with 100 monomers were applied to pack the PE matrices in three models. The detailed information for the three models is presented in Table 1. The carbon atom numbers of the two CNT/PE models were kept at approximate values (Table 1).

To establish the CNT/PE models, the PE chains were packed into the simulation box with the CNT being placed in the middle of the box. After the initial packing, two CNT/PE models underwent a 3-stage equilibrium operation, with the reference to various simulations of CNT/polymer nanocomposites [33,34,35]. The detailed process is presented as follows:

(1) Initial structure optimization at low temperature: The system was put into a canonical ensemble (NVT) for 100 ps at the temperature of 0.5 K. At this stage, the position of the CNTs was fixed. This enabled the polymer segments to have an initial position adjustment and optimization. A low temperature limits the corresponding interaction forces, which enables the entire system to have step-by-step adjustment without oversized movements of atoms.
(2) Annealing process with the temperature rising to 800 K: Under an isothermal−isobaric ensemble (NPT), a gradually increasing temperature from 0.5 K to 800 K was applied to the system within 250 ps. After reaching 800 K, the system kept running for 100 ps at a constant temperature of 800 K. For the final step, the system was cooled down from 800 K to the required temperature for a period of 250 ps. In this process, the simulation box was adjusted to the corresponding sizes under the control of pressure, and the morphology of the polymer chains was optimized to a lower energy state with the assistance of the large thermal vibrations at a high temperature.
(3) Final equilibrium process with fixed temperature: With the unchanged ensemble, the system came to an equilibrium state at the required temperature for a long period of 1 ns. At this stage, the constraints of CNTs were removed. The polymer chains kept adjusting their morphology with additional interactions of CNTs. All residual stresses in the entire system were released.

With the above 3-stage equilibrium, a reasonable structure with minimized energy and a release of the initial stress of the systems was obtained for each model. The final densities of the nanocomposite systems are listed in Table 1. As for the pure PE model, a similar preparation process was used but without the existence of CNT reinforcements.

After the preparation of the simulation cells, the tensile loading process was run through a uniform expansion of the simulation box along the Z direction, with a strain rate of 1 × 10^−5^ fs^−1^. The Nosé–Hoover extended ensemble [36] was applied to keep the temperature at 300 K (room temperature) and the constant pressure at 1.0 atm. As a result, the simulation box had a Poisson’s ratio shrinkage along the other two directions when the uniaxial strains were applied in the loading direction. In this study, interatomic interactions were described by the polymer consistent force field (PCFF) [37], which has been applied in many investigations of CNT/PE nanocomposites [13,38] and pillared graphene-reinforced PE nanocomposites [25,26]. All simulation processes were achieved in the Large-scale Atomic/Molecular Massively Parallel Simulator (LAMMPS), which is an open-source code platform with a high computation efficiency [39].

## 3. Results and Discussions

### 3.1. Reinforcement Efficiency of the CNT Junction Compared to Pure PE

**Figure 2:**
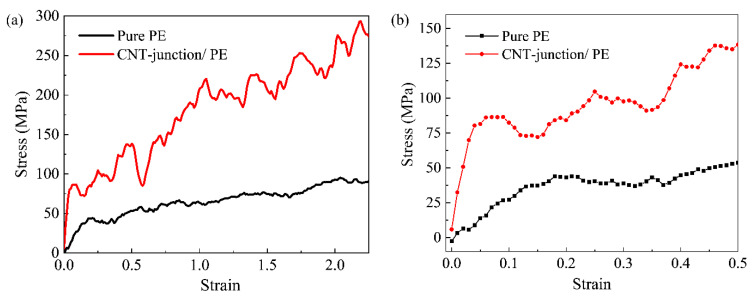
Stress–strain curves of pure PE and CNT-junction reinforced PE nanocomposite. (**a**) comparisons with strain range up to 2.5; (**b**) comparisons at small strain stage before 0.5.

The tensile performance of the CNT-junction/PE nanocomposite was compared to that of pure PE. Tensile stress–strain curves with an overall strain of up to 200% are shown in Figure 2a. The small strain stage is depicted in Figure 2b. It can be seen that the addition of connected CNTs significantly improves the overall mechanical performance of the PE matrix, including Young’s modulus, yield strength, and toughness.

The Young’s moduli of the pure PE model and the CNT-junction-reinforced PE nanocomposite were obtained via a linear fitting before the strain of 0.05. Although there were some small local fluctuations in the pure PE model, it is still reasonable to approximate it as being linear in an overall view. The Young’s moduli were calculated to be 0.28 GPa and 1.52 GPa for the pure PE model and the CNT-junction/PE model, respectively. It is known that the Young’s modulus of the PE and PE nanocomposites would vary with many parameters including temperature, strain rate, chain length of PE, and the volume fraction. Based on this fact, the obtained values of the Young’s modulus were generally in good accordance with previous studies [14,27,40]. Compared to pure PE, the CNT-junction/PE nanocomposite had a significantly higher Young’s modulus, with an increment of about 400%. Similar improvements were also concluded in a previous study on the CNT junction [30], in which the Young’s modulus of the CNT-junction/PE model was 5.3 times that of the pure weaved PE.

Apart from Young’s modulus, the yield strength of the PE matrix also rose remarkably after the addition of the CNT junction, which increased from 43.7 MPa to 86.4 MPa. The increments were as much as 100%. Moreover, the yielding point moved to a smaller strain when the CNT junction was added. The yielding strain of pure PE was about 0.17 in this study. According to a previous study on pure PE [41], the yielding strain of pure PE changes with the temperature, loading rate, and the chain length, but it is generally near the value of 0.15 at 250 K. Unlike the pure PE model, the yielding strain of the CNT/PE model was smaller than 0.05. The yielding mechanism of the polymer is often explained as the initial movement and local slips of polymer chain segments. After adding the connected CNTs, interface areas are introduced into the composite, which would increase the possibility of slippage for polymer segments near the interface. In addition to the larger stress level, the PE chains in the CNT-junction model tend to begin the interfacial slippage at an earlier time, especially at the interface area.

**Figure 3:**
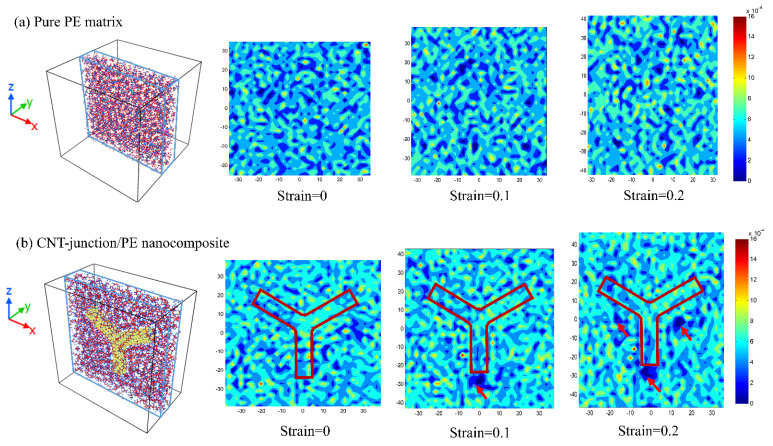
(**a**) Density distribution of pure PE at various strains. (**b**) Density distribution of CNT-junction-reinforced PE nanocomposite at various strains. (Corresponding 15 Å-layers taken from central areas are shown on the left. The red arrows point out the most remarkable local voids in the CNT-junction/PE nanocomposite.

From the density distribution in Figure 3, the evidence for the slippages of the polymer segment can be easily identified. For more explicit illustrations of the interface state, central layers of the two models with a thickness of 15 Å are taken. In the density cloud chart, the value of density is obtained by dividing the local number of atoms by the total number of atoms. For the pure PE matrix, the density distribution is relatively uniform under the same strain state and still stays stable at the strain of 0.2. However, in the model of the CNT-junction/PE nanocomposite, obvious voids can be observed near the interface. At the strain of 0.1, a remarkable void can be seen for the CNT-junction model at the lower end of the vertical CNT unit, indicating the slippage at the interface. At the strain of 0.2, two new voids appear, and they are located at the lower areas near two inclined CNTs instead of being exactly at the interface. This may be due to the more effective load transfer between inclined CNTs and the surrounding polymer chains compared to vertical CNTs. With the Z-direction tensile loading, the interfacial stress transfer between the vertical CNT unit and the polymer chains is mainly in the form of shear stresses, which can easily generate shear slippages. In the case of the inclined CNTs, however, the normal stress is much more difficult to overcome, which leads to the slippages of the polymer chains at non-interface areas.

**Figure 4:**
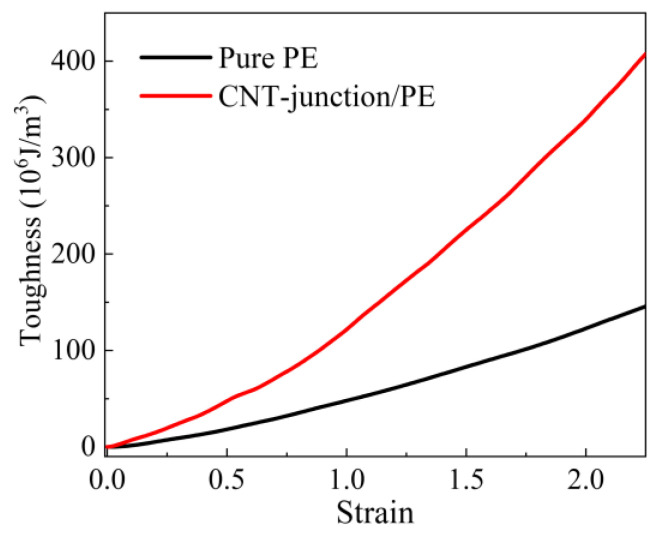
The toughness of the CNT-junction/PE model and ordinary CNT/PE model by integrating stress–strain curves in terms of the tensile strain.

Apart from the distinct properties in the early tension stage, there is also an observable difference in the slope of the two curves for the large strain stage. Both models begin to have a strain-hardening stage after the yielding, but the slope of the CNT-junction model is remarkably larger than that of the pure PE model. As a result, there is a great enhancement of toughness after the introduction of the CNT junction, which can be seen from the sizes of the area enclosed by two curves. To quantitatively characterize the toughness increment, we follow the approach used by Gersappe [42] and integrate the tensile curves to obtain the dissipated work curves in terms of the increasing strain. It can be seen that the values in the two curves rise as the strains increase, but the CNT-junction/PE nanocomposite possesses a remarkably higher increasing rate (Figure 4). The final values reach 145.6 × 106 J/m^3^ and 407.6 × 106 J/m^3^ for the pure PE and CNT-junction/PE models, respectively, indicating the prominent toughening effect of about 200% increment. Reflecting the capability of energy dissipation, toughness is a crucial property for polymers, especially for a soft polymer with high flexibility, like PE. Hence, the CNT junction is a beneficial sub-structure for the overall mechanical properties of CNT/PE nanocomposites.

**Figure 5:**
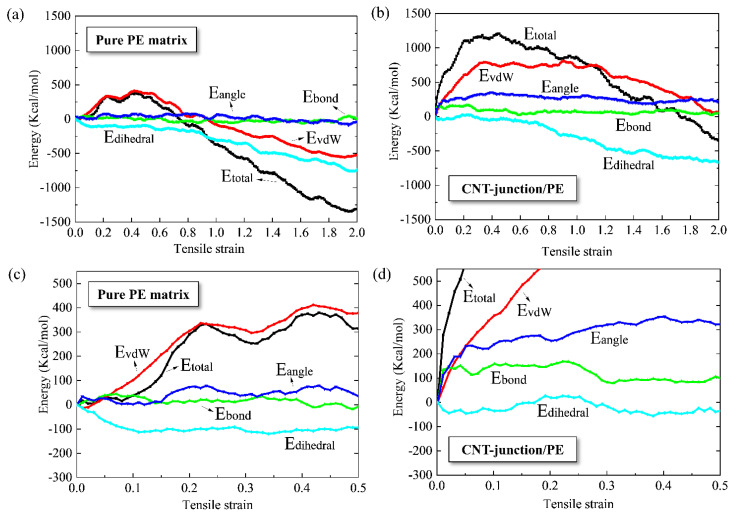
(**a**) Energy evolution curves of pure PE for overall stain range; (**b**) energy evolution curves of CNT-junction reinforced PE nanocomposite for overall stain range; (**c**) energy evolution curves of pure PE at small strain stage before 0.5; (**d**) energy evolution curves of CNT-junction reinforced PE nanocomposite at small strain stage before 0.5.

According to previous studies, the reinforcement mechanism of CNT/polymer nanocomposite is generally concluded to be a denser packing of the polymer matrix due to the enhanced interfacial interactions [43]. Apart from that, more detailed information can be provided according to the analysis of the energy evolution. The energy evolution curves of the pure PE and the CNT-junction/PE model were compared, including the total potential energy, vdW energy, bond energy, angle energy, and dihedral energy. The corresponding results are illustrated in Figure 5.

In general, there are both similarities and differences between the energy variations of the CNT-junction/PE and pure PE models. The bond, angle, and dihedral energy variations of the pure PE and the CNT-junction/PE are very close, while the differences between vdW energies are significant. It can be seen that there is a 750 Kcal/mole increase in the vdW energy in the tension of the CNT-junction/PE nanocomposite, but the increment for pure PE is less than 500 Kcal/mole.

There are three parts that may contribute to the vdW energy of the nanocomposite: PE matrix interaction, CNT junction internal interaction, and interfacial interaction. The internal vdW interactions of CNTs are very small due to the limited atom number. Moreover, the atom number of the PE matrix in the CNT-junction/PE model is about 25% less than that of the pure PE model, which is not able to contribute more vdW energy increments. Hence, it is the enhanced interfacial interactions between the CNT junction and the PE matrix that cause such significant increments of the vdW energy in the nanocomposite system. This conclusion is in accordance with previous studies on ordinary CNT-reinforced PE nanocomposites [43,44].

There is another noticeable difference between pure PE and the CNT-junction/PE nanocomposite in Figure 5. For pure PE, the variations in total potential energy overlap with the curves of the vdW energy at the early tension stage. In contrast, the total potential energies of the CNT-junction/PE nanocomposite are much greater than the vdW energies at the initial states. Such difference is mainly related to the contribution of bond and angle energies. It can be seen that the bond and angle energies of pure PE remain almost unchanged throughout the loading process. For the CNT-junction/PE nanocomposite, however, there are considerable increments in bond and angle energies at the very early tension stage.

During the tension of the CNT/PE nanocomposite, it is known that the topological deformation of the CNT is rather limited due to its extremely high elastic modulus [40]. Thus, it is the structural evolution of PE chains that results in the bond and angle energy variations in the CNT/PE nanocomposite system, which are not seen in the pure PE model. Such difference is induced by the different strengths of the interactions in the CNT-junction/PE model and the pure PE model. Slippages happen easily in the pure PE model due to the weaker interactions between polymer chains, which, accordingly, disable the elongation of polymer backbones. Yet the stronger interfacial interactions of the CNT-junction/PE nanocomposite mean that stress can be effectively kept at the perimeter zone of reinforcements. Accordingly, the bond and angle deformation could occur in a partial group of polymer segments.

### 3.2. Comparisons of the Tensile Performance between the CNT-Junction/PE Nanocomposite and the Ordinary CNT/PE Nanocomposite

**Figure 6:**
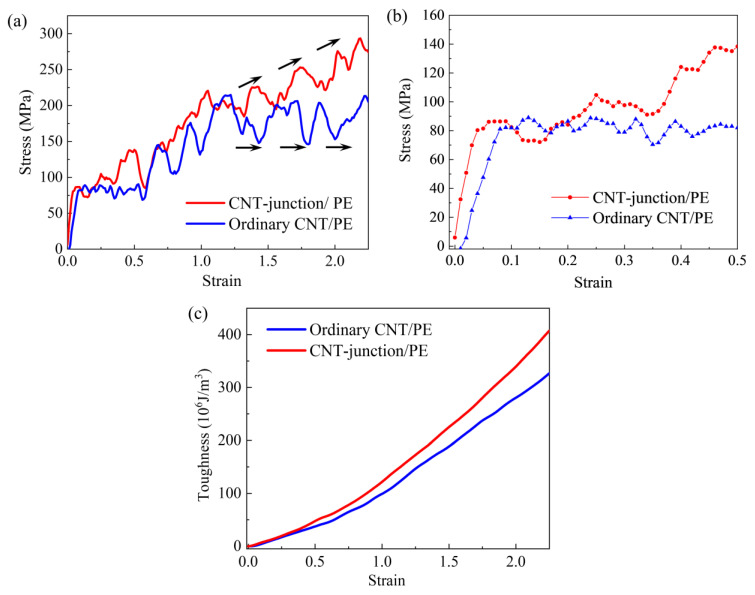
(**a**) Stress–strain curves of two different CNT/PE nanocomposite models in the strain range of up to 2.5 (black arrows display curve directions); (**b**) stress–strain curves of two different CNT/PE nanocomposite models at small strain stage; (**c**) toughness comparisons between CNT-junction/PE model and ordinary CNT/PE model.

To further identify the unique reinforcing effect of the CNT junction, the CNT-junction PE nanocomposite is also compared with the ordinary CNT/PE nanocomposite (Figure 6). Tensile stress–strain curves with a strain of up to 2.5 are shown in Figure 6a. The small strain stage is presented in Figure 6b.

Although the differences between the CNT-junction model and the ordinary CNT model are not as prominent as the enhancement in the pure PE matrix, the better performances induced by the CNT junction are still considerable. From Figure 6b, it can be seen that the CNT-junction model has a higher Young’s modulus than the ordinary CNT model at the linear tensile stage. The Young’s modulus of the ordinary CNT model is calculated to be 1.1 GPa. Thus, the increment in the Young’s modulus caused by the CNT junction is about 60%.

The CNT-junction/PE model has a competitive yield strength as the ordinary CNT model. This is easy to understand since the yielding of both the CNT-junction model and the ordinary CNT model is generated from the initial interfacial slippage. A similar shear-stress-dominated CNT/PE interface exists in both the ordinary CNT model and the CNT-junction model (vertical CNT unit and the matrix).

Furthermore, continuing to the strain hardening stage, the tensile curves of the CNT-junction/PE model present different trends from the ordinary CNT/PE model, especially after the strain of 1.5 (Figure 6a). Although there are some fluctuations, the tensile curve of the CNT-junction/PE model is still an upward growth trend after the yielding point. However, the tensile stress of the ordinary CNT/PE model seems to have a plate-style fluctuation in a stable range. Due to this difference, the CNT-junction/PE model possesses a better performance in toughness than the ordinary CNT/PE model, which is presented in Figure 6c. It can be seen that the toughness of the CNT-junction/PE model is higher than that of the CNT/PE model after the strain of 0.5. As the strain increases to 2.5, the CNT junction causes about a 25% increment in toughness, in total, compared to the ordinary CNT/PE nanocomposite.

**Figure 7:**
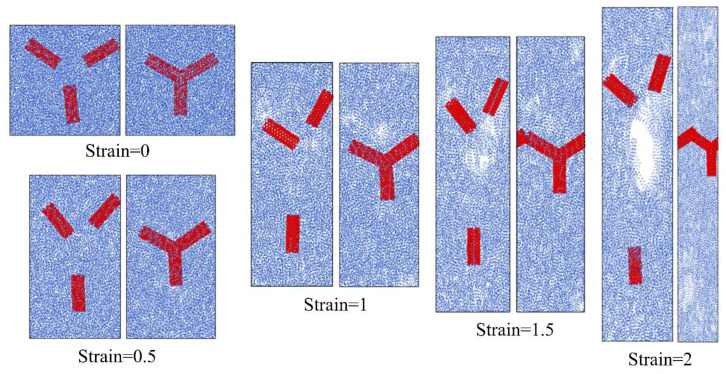
Snapshots for the CNT-junction/PE model (**right**) and ordinary CNT/PE model (**left**) under different strains.

Combined with the snapshots in Figure 7, the tensile performance differences between the CNT-junction/PE model and the ordinary CNT/PE can be better identified. During the early tension stage before a strain of 0.5, there is no significant difference in the polymer matrix state between the two models. However, there is a distinct difference in the state of the CNTs. The ordinary CNT/PE model shows a noticeable translation and rotation of individual CNTs, whereas the location of the CNT junction remains very stable in the CNT-junction/PE model.

As the tensile strain increases, slippages are generated in between polymer chains as well as on the interface, leading to the generation and propagation of the voids. It should be noted that the void states of the two models tend to be totally different. Compared to the CNT-junction/PE model, more and larger voids are generated in the ordinary CNT/PE model. At a strain of 2.0, very large pores throughout the entire section can be seen for the ordinary CNT/PE model, but there is no conspicuous pore in the CNT-junction model for the same strain states.

The generation and propagation of the voids are basically related to the mobility behavior of the CNT in the polymer matrix. In the ordinary CNT/PE model, individual CNTs are easy to move with the flow of the PE matrix. Accordingly, the polymer chains at the central area in between three individual CNTs are the most stretched ones, and the decisive voids are eventually generated in this place. By contrast, the immobility of the CNT junction leads to a slower progression of the PE chain deformation, as well as the generation of the voids. A previous investigation also concluded that the toughening efficiency of the CNT/PE nanocomposite is related to the mobility of the reinforcements [45].

**Figure 8:**
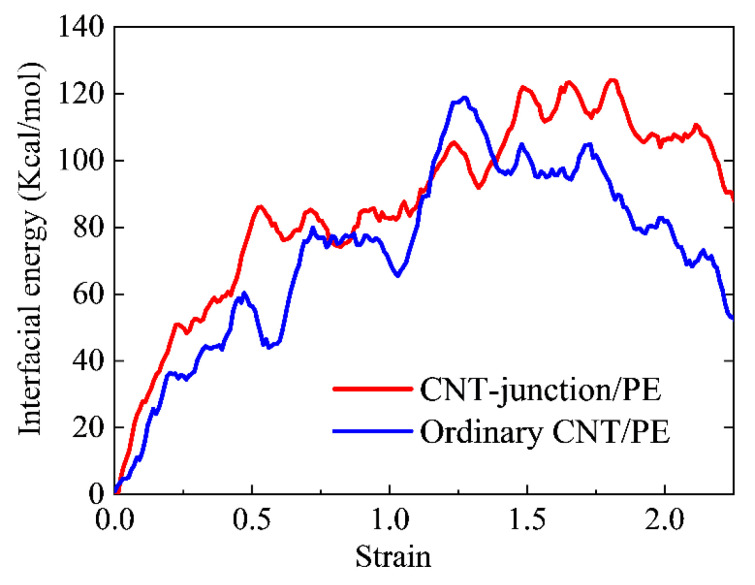
Comparisons between the CNT-junction/PE model and ordinary CNT/PE model in terms of variations of interaction energy.

In Figure 8, the variations in interaction energy are presented for both the CNT-junction/PE model and the ordinary CNT/PE model. It can be seen that, most times, the CNT-junction/PE model has greater values of interaction energy than the ordinary CNT/PE model. Corresponding to the tensile curves, the larger interaction energy between the CNT junction and PE matrix leads to an improvement in Young’s modulus. In addition, different reductions in interaction energy after the strain of 1.5 are in accordance with the different curve trends of the two models in tensile curves. The greater reductions in interfacial energy in the ordinary CNT/PE model also correspond to the larger voids in the snapshots of the ordinary CNT/PE model in Figure 7.

The reason behind the greater interfacial interaction energy of the junction is related to the Stone–Wales (SW) defects in the connection area, which have been proven to have a profitable effect on the interfacial performance of polymer matrix and reinforcements. Li and coworkers [46] concluded that SW defects could enhance the interfacial strength of the graphene/epoxy composites. Yang [47] reported that the Young’s modulus of the CNT/PE nanocomposite is related to the existence of SW defects. In the simulations of Alian and Meguid [21], the pull-out force of models with SW defects was significantly increased compared with the straight CNTs.

In summary, the CNT junction has a better core effect than the ordinary CNT in the PE matrix, which can not only densify the PE matrix but also ensure that the polymer nanocomposite has a stable deformation framework. The limited mobility of the junction geometry impedes the expansion of voids in the PE nanocomposite and provides a larger interfacial interaction energy, resulting to a better mechanical performance of the PE matrix. Therefore, it is beneficial to create more connections between individual CNTs or apply CNT-based networks in the applications of polymer nanocomposites.

## 4. Conclusions

In this study, a CNT junction was added to a PE matrix as a new reinforcement. Using MD simulations, the mechanical properties of the CNT-junction/PE nanocomposite were investigated and evaluated by comparing them with those of the pure PE matrix and the ordinary CNT/PE nanocomposite. The following conclusions are obtained from the previous discussions.

The addition of the CNT junction significantly improves the mechanical properties of the PE matrix, including Young’s modulus and toughness, for which the corresponding increments are at least 200%. The CNT-junction brings a 200% increase in the yield strength of the PE matrix but makes the yielding happen at an early strain state, because the introduction of the interface accelerates the initial slippage of the polymer chains. Based on the analysis of the potential energy, it is found that the addition of the CNT junction significantly increases the vdW interactions. Moreover, polymer chains tend to have more deformation contributions from the aspect of bond and angle.

The CNT junction has a better reinforcement efficiency for the PE matrix than the ordinary straight CNT. The increments in the Young’s modulus and toughness are as much as 60% and 20%, respectively. The major improvements in the CNT junction are due to the limited mobility and enhanced interfacial energy. On the one hand, the limited mobility of the CNT junction slows down the void propagation of the PE matrix. On the other hand, the enhanced interfacial energy makes the matrix denser. The increases in the interfacial energy are essentially related to the Stone–Wales defects, which appear in the connection of the CNT junction.

To summarize, CNT junctions are beneficial structures for the mechanical performance of CNT/PE nanocomposites. The conclusions of this study could provide some valuable information for the design and application of CNT/PE nanocomposites.

## Figures and Tables

**Table 1:** Parameters of two CNT/PE models with ordinary CNT and CNT junction.

Models	CNT Atoms	Matrix Atoms	Total Atoms	Final Density at 300 K (g/cm^3^)
CNT-junction/PE	C 798 H 36	C 10,000 H 20,100	30,934	0.854
Ordinary CNT/PE	C 792 H 72	C 10,000 H 20,100	30,964	0.850
Pure PE	0	C 12,800 H 25,728	38,528	0.80

## Data Availability

Data are contained within the article.
